# Converting Redox Signaling to Apoptotic Activities by Stress-Responsive Regulators HSF1 and NRF2 in Fenretinide Treated Cancer Cells

**DOI:** 10.1371/journal.pone.0007538

**Published:** 2009-10-21

**Authors:** Kankan Wang, Hai Fang, Dakai Xiao, Xuehua Zhu, Miaomiao He, Xiaoling Pan, Jiantao Shi, Hui Zhang, Xiaohong Jia, Yanzhi Du, Ji Zhang

**Affiliations:** 1 State Key Laboratory of Medical Genomics and Shanghai Institute of Hematology, Ruijin Hospital affiliated to Shanghai Jiao Tong University School of Medicine (SJTU-SM), Shanghai, China; 2 Key Laboratory of Stem Cell Biology, Institute of Health Sciences, Shanghai Institutes for Biological Sciences (SIBS), Chinese Academy of Sciences (CAS) and SJTU-SM, Shanghai, China; 3 Laboratory of Translational Research (LTR), Sino-French Center in Life Sciences and Genomics, Ruijin Hospital affiliated to SJTU-SM, Shanghai, China; Istituto Dermopatico dell'Immacolata, Italy

## Abstract

**Background:**

Pharmacological intervention of redox balance in cancer cells often results in oxidative stress-mediated apoptosis, attracting much attention for the development of a new generation of targeted therapy in cancer. However, little is known about mechanisms underlying the conversion from oxidative signaling to downstream activities leading cells to death.

**Methodology/Principal Findings:**

We here report a systematic detection of transcriptome changes in response to oxidative signals generated in leukemia cells upon fenretinide treatment, implicating the occurrence of numerous stress-responsive events during the fenretinide induced apoptosis, such as redox response, endoplasmic reticulum stress/unfolded protein response, translational repression and proteasome activation. Moreover, the configuration of these relevant events is primarily orchestrated by stress responsive transcription factors, as typically highlighted by NF-E2-related factor-2 (NRF2) and heat shock factor 1 (HSF1). Several lines of evidence suggest that the coordinated regulation of these transcription factors and thus their downstream genes are involved in converting oxidative signaling into downstream stress-responsive events regulating pro-apoptotic and apoptotic activities at the temporal and spatial levels, typifying oxidative stress-mediated programmed death rather than survival in cancer cells.

**Conclusions/Significance:**

This study provides a roadmap for understanding oxidative stress-mediated apoptosis in cancer cells, which may be further developed into more sophisticated therapeutic protocols, as implicated by synergistic induction of cell apoptosis using proteasome inhibitors with fenretinide.

## Introduction

The development of cancer therapies can benefit from the accumulated knowledge in cancer biology, particularly with respect to cancer hallmarks such as self-sufficiency in growth signals, evasion of programmed cell death and metastasis [1]. Recent experimental and clinical data provide compelling evidence that the reduction/oxidation (redox) signaling pathways may play an essential role in carcinogenesis and malignant progression [2]. In general, malignant cells are intrinsically under pro-oxidant microenvironment, with increased steady-state levels of reactive oxygen species (ROS) [3], representing another promising component of biological differences between cancer and normal cells. Recently, new therapeutic intervention strategies producing a state of selective oxidative stress in cancer cells have gained importance [4].

Redox regulation has been shown to be an important mechanism of malignant cell survival. Shifting the cellular redox balance through pharmacologic manipulation in favor of increasing intracellular ROS may lead to oxidative stress and subsequent induction of apoptosis within cancer cells. The engagement of apoptosis in cancer cells induced by ROS-generating agents is probably accompanied by the activation of endoplasmic reticulum (ER) stress. Apoptosis can be initiated by death receptors stimulating the extrinsic pathway, or by perturbation of intracellular homeostasis involving mitochondria-associated intrinsic pathway and ER stress-mediated pathway. These initiating pro-apoptotic signals finally converge on central executioner of apoptosis by the disruption of mitochondrial transmembrane potential (MMP) in mitochondria as well as the activation of caspase cascades. Reaching the level to an extent exceeding the endurable redox threshold, ROS can act as specific signals stimulating ER stress-mediated apoptosis specifically in cancer cells. In response to various stimuli including oxidative stressors [5], [6], ER has evolved unfolded protein response (UPR) modulating several transcription factors (e.g., ATF6, XBP1 and CHOP) in an attempt to adapt for survival or otherwise undergo apoptosis facing prolonged UPR. However, there is limited knowledge about mechanisms underlying the conversion from oxidative signaling to downstream stress events leading cells to death. With the availability of appropriate therapeutic ROS-generating agents, systematic characterization of gene expression and the underlying transcriptional regulation will be the key to the elucidation of such conversion.

With the development of ROS-generating agents such as arsenic trioxide (ATO) for the treatment of acute promyelocytic leukemia (APL) [7], the possibility of exploiting selective oxidative stress as apoptosis-inducing cancer therapy is the emerging as a promising therapeutic option. Experimental data have show that the therapeutic effectiveness of ATO is mediated by ROS intracellular production and subsequent apoptosis [8]. Although ROS-inducing agents like ATO have shown great potentials in the treatment of malignant cells, the side effects remain to be fully evaluated [9]. There is considerable interest in designing the most rationale redox-active strategies with minimal *in vivo* side effects. In this aspect, N-(4-hydroxyphenyl) retinamide (fenretinide), a synthetic retinoid with several long-term clinical trials, is worthy of further investigation [10]. Unlike such natural retinoids as *all-trans* retinoic acid (ATRA), fenretinide exerts distinct biologic effects, preferentially engaging the apoptotic pathway in many tumor cells targeting ROS while maintaining its minimal *in vivo* cytotoxicity to normal cells [11], [12]. Mechanisms of fenretinide-induced apoptosis have been intensively studied [13]–[15]. Recent data suggest that this ROS-generating agent may perturb cellular homeostasis and modulate the various stress-related genes, implicating that the involvement of ROS-dependent ER stress may render the susceptibility of cancer cells to fenretinide-induced apoptosis [16]. However, the mechanisms by which ROS formation leads to ER stress and cancer cell apoptosis are far from clear. Detailed elucidation of these mechanistic links may allow insight into oxidative stress-mediated apoptosis in cancer cells and permit the optimization of cancer-specific targeting therapies.

We speculate that cancer cells with predisposition of redox signaling are most likely sensitive to oxidative stimuli from ROS-generating agents such as fenretinide, undergoing oxidative stress-mediated apoptosis. To precisely uncover regulatory mechanisms underlying the conversion from oxidative signaling to downstream stress events exerted on ER and eventually to death outcomes rather than survival advantages, we employed integrative methods of advanced data mining with microarray technology to profile transcriptome changes in a fenretinide-sensitive cell line, and found numerous temporal-spatial relationships between stress-responsive events. Moreover, stress-responsive transcription factors, as highlighted by NF-E2-related factor-2 (NRF2) and heat shock factor 1 (HSF1), play prominent roles in the configuration of these relevant events. Validations through immunofluorescene and chromatin immunoprecipitation assays and stress-related transcriptome comparisons further provided evidence that these stress-responsive regulators and thus their target genes are involved in converting oxidative signaling into downstream stress activities including redox response, ER stress/UPR and proteasome activation, representing typical events of oxidative stress mediated apoptosis in fenretinide-treated malignant cells.

## Results

**Figure 1:**
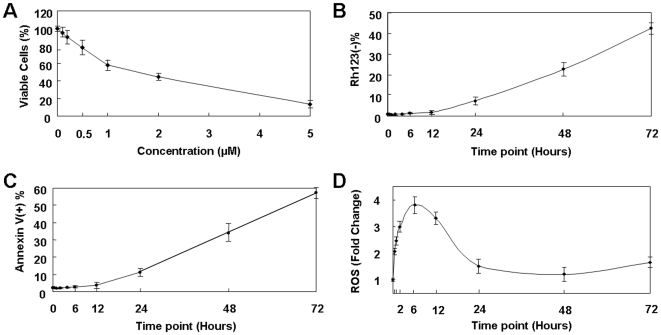
Cellular and molecular characterization of fenretinide-induced apoptosis in leukemia-derived NB4 cells. (A) Cell viability was evaluated using an MTT assay after various doses of fenretinide for 48 hours. (B) Loss of mitochondrial membrane potential Δ_Ψm_ with 1 µM fenretinide treatment, as determined through rhodamine 123 and propidium iodide (PI) double staining, and followed by flow cytometry analysis. (C) Apoptosis after 1 µM fenretinide treatment was evaluated by annexin V-specific antibody and PI double staining and flow cytometry analysis. (D) Dynamic changes of ROS, as evaluated in cells stained with DCFH-DA and followed by flow cytometry analysis. Mean values±SD are plotted from three independent experiments.

Fenretinide induces intracellular production of ROS and thus apoptosis in a variety of malignancies including leukemia [10]. We analyzed the antiproliferative and apoptotic effects of fenretinide on leukemia-derived cell lines NB4, U937, and HL60, and found that these cell lines underwent growth inhibition and apoptosis in response to 1–2 µM of fenretinide, and that their susceptibilities appeared to be correlated with levels of ROS (Supplementary Figure S1). Based on its relatively high sensitivity to drug-induced ROS generation and apoptosis, NB4 was chosen as a prototype cell line for cellular and molecular assessments prior to detailed transcriptome analysis. As shown in Figure 1A, NB4 cell growth was inhibited by fenretinide treatment in a dose-dependent manner. Treatment with a low dose (1 µM) of fenretinide appeared to be sufficient to induce apoptosis in NB4 cells within 72 hours, as shown by mitochondrial membrane potential and annexin V assays (Figures 1B and 1C). We further examined intracellular ROS changes during this time course. Surprisingly, we found that ROS changes were more complex than previously recognized, displaying left-skewed bell-shape curve (Figure 1D). As expected, ROS accumulated sharply, reaching a four-fold increase compared to the basal levels within 6 hours of treatment, whereas it unexpectedly decreased gradually thereafter to levels corresponding roughly to twice the basal levels of untreated cells. This data suggests the involvement of redox signaling in NB4 cells upon fenretinide treatment. Fenretinide stimulation causes a rapid accumulation of intracellular ROS, which may in turn activate cellular mechanisms to reduce ROS levels. Also, the moderate levels of intracellular ROS are probably required for fenretinide-induced apoptosis.

### Robust transcriptome profiling of fenretinide-induced apoptosis

#### Time-series microarray hybridization, gene selection, and identification of transcriptome features

**Figure 2:**
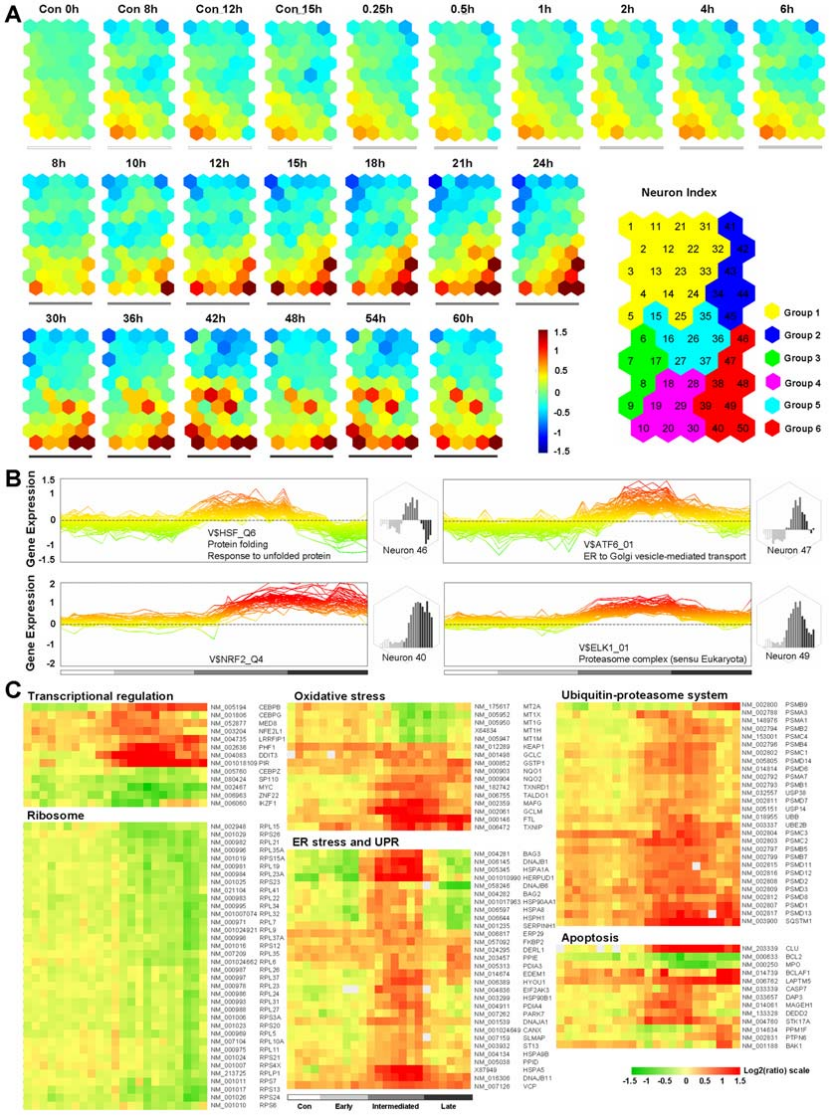
In-depth analysis of transcriptome changes induced by fenretinide in NB4 cells. (A) Illustration of transcriptome changes by CPP-SOM. Each presentation illustrates a time-point specific transcriptome map, in which all the up-regulated (represented by neurons in red), down-regulated (represented by neurons in blue) and moderately regulated (represented by neurons in yellow and green) genes are well delineated. Color bar stands for expression values (log ratio with base 2), with brighter to denote the higher value. Presentations in control series are indicated by white bar underneath, while those in fenretinide-treated series are partitioned into three stages: early, intermediate and late, as indicated by gray-graded bar underneath. All the presentations are linked by positions, i.e., the same position represents the same neuron whose index is shown in the enlarged grid ideogram on the right panel. Six recognizable regions obtained through hierarchical clustering based on pattern similarities are colored coded as indicated. Genes in Group 6 are most prominently up-regulated during the apoptosis, representing an oxidative stress-responsive transcriptome signature spectrum. (B) Illustration of expression patterns of genes in representative neurons of Group 6 through color-coded line graphs and bar charts, as exemplified by neurons 46, 47, 40 and 49. Their corresponding PWM and/or GO enrichments are also indicated. (C) Major functional features associated with oxidative stress-mediated apoptosis, as visualized by hierarchical clustering of representative genes.

To analyze the detailed mechanisms underlying fenretinide-induced apoptosis, we performed transcriptome profiling on samples of fenretinide-treated NB4 cells which were collected at 19 time points and untreated cell samples at 4 time points. After microarray hybridization and data acquisition, gene expression data were first subjected to a topology-preserving gene selection procedure through self-organizing map (SOM) integrated singular value decomposition (SVD). Following the procedure based on false discovery rate (FDR) statistical inference, a total of 3,345 regulated genes with characteristic patterns were selected (see Methods) and further analyzed by component plane presentation (CPP) integrated SOM [17]–[19]. As shown in Figure 2A, each presentation illustrates a timepoint-specific transcriptome map, permitting direct comparisons of transcriptome changes within/between the control series and the fenretinide-treated series. Comparing the control and treatment series, the observed transcriptome changes before the 6-hour treatment (termed the early stage) are mostly due to culture duration, implying that early-stage effects induced by fenretinide are mainly biochemical, with limited effects on transcriptional regulation. However, prominent transcriptome changes become apparent after 8 hours of treatment, as highlighted by genes mapped to neurons in bottom-right corners (also termed Group 6 on the right panel of Figure 2A). These genes are prominently up-regulated after the early stage, representing a major transcriptome feature during fenretinide-induced apoptosis. Since ROS accumulation is a prominent effect of fenretinide treatment, it is logical to speculate that modulation of these genes is a result of ROS accumulation.

#### Transcriptional and functional features of clustered genes characteristically highlighting oxidative stress-mediated apoptosis

Our robust transcriptome approach allows the clustering of genes with highly similar expression patterns into the same or nearby neighboring neurons, as illustrated in Figure 2B. This may facilitate many aspects of in-depth mining of biological information relevant to the fenretinide-induced apoptosis. We performed transcription factor binding site (TFBS) enrichment analysis following hypergeometric distribution-based multiple hypothesis tests to infer common transcriptional features of clustered genes (see Methods). As illustrated by representative neurons of Group 6 (Figure 2B), the transcription factors NRF2, HSF1, ATF6 and ELK1 are significantly over-represented respectively in neuron 40, 46, 47 and 49. NRF2 is known to activate transcription of genes encoding antioxidative proteins under oxidative stress [20], [21], HSF1 is a transcription factor responsible for expression of heat shock genes [22], ATF6 is a key transcriptional activator of unfolded protein response (UPR) [23], and ELK1 is involved in transcription of survival genes [24]. These data suggest that the genes in Group 6 are largely regulated by stress-responsive transcription factors, highlighting the impact of upstream oxidative signaling on downstream effects.

To further address functional importance of clustered genes, we employed Gene Ontology (GO) for functional enrichment analysis. Functional features with statistical significance were revealed, depicting a relatively comprehensive view of oxidative stress-mediated apoptosis. Among these features were genes involved in transcriptional regulation, ribosome machinery, oxidative stress, ER stress/UPR, ubiquitin-proteasome system, and apoptosis (Figure 2C). Changes of genes involved in transcriptional regulation appear to be logical for the admission of malignant cells into programmed cell death, as indicated by up-regulated *DDIT3*/*CHOP*, *CEBPB*, *CEBPG*, *NFE2L1* and *PHF1*, and down-regulated *MYC* and *IKZF1*. Reduced ribosome activity may represent a direct response to stress-repressed overall protein translation. Regulation of redox-related genes may account for ROS reduction during later stages of fenretinide-induced apoptosis. Up-regulation of a large number of ER stress- and UPR-regulated genes was observed during the period from 6 to 24 hours after treatment (termed the intermediate stage), implicating the occurrence of ER stress- and UPR-related defense activities. Notably, we observed activation of genes involved in the ubiquitin-proteasome system. Most of genes coding for the proteasome apparatus were induced after the early stage, promoting the degradation of overloaded unfolded/misfolded proteins resulting from ER stress/UPR. Up-regulation of genes encoding regulators/participants of apoptotic cascades (e.g., *CASP7*, *BCLAF1*, *DEDD2*, *DAP3*, *STK17A*, *LAPTM5* and *MAGEH1*) and down-regulation of negative apoptosis regulators such as *BCL2* and *MPO* were apparent during the intermediate and late stages.

### Additional molecular and cellular evidence for oxidative stress-mediated apoptosis in fenretinide-treated leukemia cells

#### The sequential involvement of ER stress/UPR and mitochondria associated apoptotic activities

**Figure 3:**
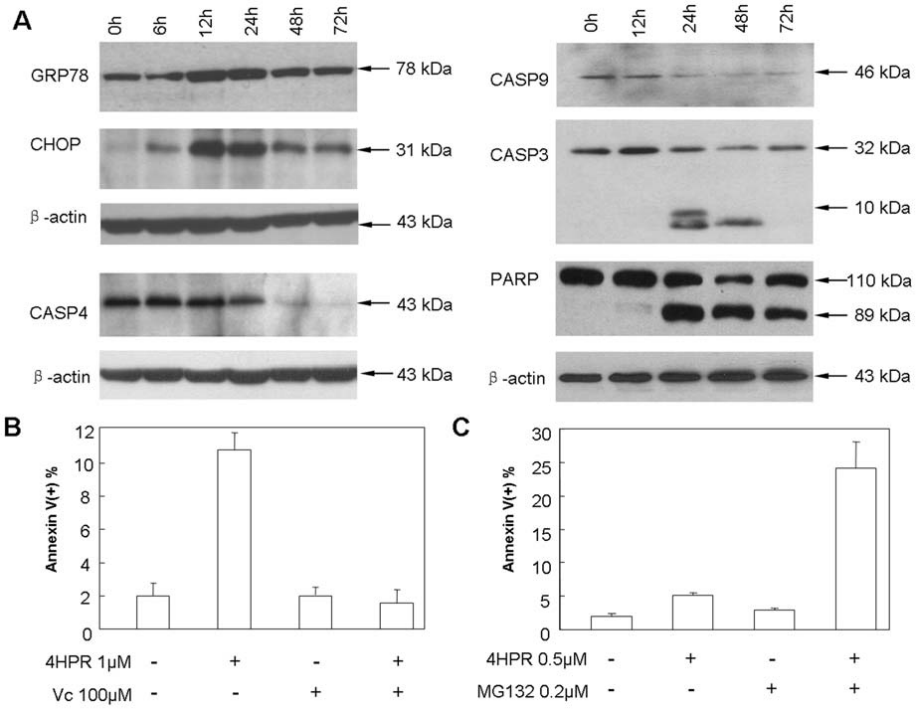
Cellular and molecular evidence of oxidative stress-mediated apoptosis in fenretinide treated cells. (A) Western blot analysis of ER stress/UPR related markers and apoptotic caspases upon 1 µM fenretinide treatment in NB4 cells. (B) Abrogation of fenretinide-induced apoptosis by vitamin C. (C) Synergistic induction of cell apoptosis by proteasome inhibitor MG132 and fenretinide. Apoptosis was evaluated by annexin V-specific antibody and PI double staining and flow cytometry analysis. The results represent the average of three independent evaluations ± SD.

To validate features revealed by transcriptome analysis and to identify additional components of oxidative stress-mediated apoptosis, we further conducted a series of cellular and molecular assays. As shown in the left panel of Figure 3A, changes in protein levels of the ER stress/UPR marker GRP78/HSPA5 and the stress-inducible pro-apoptotic transcription factor CHOP/GADD153 were correlated with mRNA levels (Figure 2C). These genes and proteins were specifically up-regulated during the intermediate stage, providing further evidence that ER stress/UPR occurred during this time frame. Additionally, the pro-apoptotic form of CASP4, a ER stress-specific caspase [25], was dramatically reduced at the late stage, implicating the involvement in fenretinide-induced apoptosis. As shown in the right panel of Figure 3A, mitochondria associated apoptotic caspase cascades were activated at the late stage. Pro-apoptotic CASP9 was reduced, whereas CASP3 was increased in its active form prior to the late stage. Moreover, cleaved PARP was observed following caspase cascade activation. In sum, the protein biochemical data also support the notion that ER stress/UPR occurs at the intermediate stage, while mitochondria-involved apoptosis occurs mainly at the late stage.

#### Synergistic induction of cell apoptosis by fenretinide and proteasome inhibitor

In fenretinide-treated cells, ROS signaling may represent an essential stimulus at the early stage of programmed cell death. To provide further evidence for ROS signaling in apoptosis, we performed an antagonist assay using vitamin C as the antioxidant. As shown in Figure 3B, vitamin C treatment completely abrogated fenretinide-induced apoptosis in NB4 cells. Genes encoding proteasome components were significantly up-regulated during the intermediate and late stages. Thus, we hypothesized that proteasome activity might function as a defense mechanism coupled to the UPR for unfolded/misfolded protein degradation to reduce the ER stress burden [26]. Accordingly, proteasome activation may antagonize the pro-apoptotic/apoptotic cascade. To explore this hypothesis, we used the proteasome inhibitor MG132 to block proteasome activity during fenretinide-induced apoptosis. As shown in Figure 3C, a sub-cytotoxic concentration (0.2 µM) of MG132 together with a low dose of fenretinide (0.5 µM) induced significant cell apoptosis within 48 hours, demonstrating synergistic rather than antagonistic effects of the two compounds.

### Converting oxidative signaling into downstream effects through stress-responsive transcription factors as highlighted by NRF2 and HSF1

#### Coordination between temporal-spatial changes of NRF2 and HSF1, and expression patterns of their potential target genes

**Figure 4:**
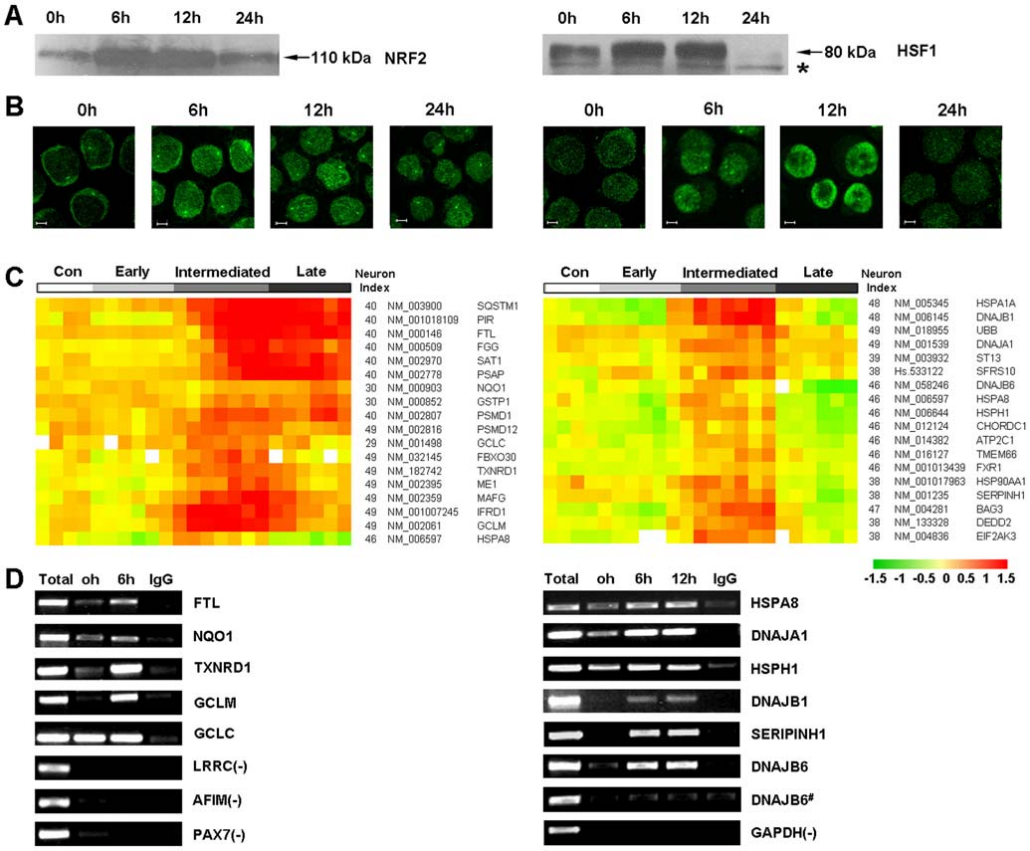
Coordinated regulation of stress-responsive transcription factors, i.e., NRF2 and HSF1, and their target genes. (A) Western blot analysis of NRF2 and HSF1 from nuclear extracts of NB4 cells untreated or treated with 1 µM fenretinide at the indicated time points. “*” indicates the non-specific binding band. (B) Nuclear translocation of NRF2 and HSF1 following 1 µM fenretinide treatment in NB4 cells, as visualized by immunofluorescence microscopy (scale bars, 5 µm). (C) Illustration of expression patterns of genes potentially targeted by NRF2 and HSF1, illustrated in the left and right panel, respectively. (D) ChIP combined with PCR assays to validate the physical interaction between transcription factors (i.e., NRF2 and HSF1) and their target genes. Total: total input; IgG: ChIP reaction with IgG antibody as a control; DNAJB6^#^: primers designed from non-TFBS region of the gene *DNAJB6*.

Our robust transcriptome profiling approach facilitated the in-depth mining of biological information relevant to oxidative stress-mediated apoptosis, including the prediction of upstream transcription factors involved in gene regulation. Of transcriptional regulators predicted, the stress-responsive transcription factors NRF2 and HSF1 are of particular interest for understanding how oxidative signaling is translated into downstream effects. We therefore further investigated the temporal abundance and spatial localization of these two stress-responsive transcription factors during apoptosis. As demonstrated in Figure 4A, protein levels of both NRF2 and HSF1 were markedly elevated in nuclear extracts within 6 hours of exposure to fenretinide, and their temporal abundance was differentiated thereafter. NRF2 induction was extended beyond 24 hours whereas HSF1 induction was terminated at this time point. Similarly, immunofluorescence microscopy analyses revealed marked accumulation of both factors in nuclei of cells treated with fenretinide for 6 hours, compared to a diffuse distribution of NRF2 and HSF1 in untreated cells (Figure 4B). Also, nuclear gathering of NRF2 was sustained beyond the 24 hour treatment period whereas that of HSF1 was terminated. Considering the relatively low levels of ROS at 24 hour treatment (Figure 1D), the inactivation of HSF1 is probably due to a reducing microenvironment [22]. Moreover, the temporal-spatial changes of NRF2 and HSF1 correlate well with regulatory patterns of their potential target genes (Figure 4C). Up-regulated expression of NRF2 potential target genes was extended to the late stage, whereas gene expression of HSF1 potential targets was unanimously terminated by the end of the intermediate stage.

#### Physical interactions between NRF2 and HSF1, and their target genes upon activation

To explore whether NRF2 and HSF1 are physically bound to their targets, we conducted chromatin immunoprecipitation (ChIP) assays using antibodies against NRF2 or HSF1. Based on the predicted TFBS of the representative genes listed in Figure 4C, specific PCR primers were designed using ChIP products of either NRF2 or HSF1 as DNA templates. As illustrated in the left panel of Figure 4D, genes with the predicted TFBS of NRF2 (i.e., *FTL*, *NQO1*, *TXNRD1*, *GCLM* and *GCLC*) are positive for NRF2 ChIP products, whereas unrelated genes (i.e., *LRRC*, *AFIM* and *PAX7*) are negative in the same products. Although a basal level of NRF2 binding was observed in untreated ChIP products, most of the predicted genes revealed significantly stronger signals in treated samples. Likewise, ChIP-PCR assays of HSF1 revealed similar results (Figure 4D, right panel). Notably, primers designed from the TFBS region revealed prominent bands in HSF1 ChIP products, whereas those from the non-TFBS regions of the same genes revealed absent signals (e.g., *DNAJB6 vs. DNAJB6^#^*). Altogether, our evidence indicates that NRF2 and HSF1 are activated upon ROS accumulation by the end of the early stage, converting oxidative signaling into downstream effects by directly acting on their target genes. NRF2 activation extends to the late stage, while HSF1 activity is terminated by the end of the intermediate stage.

#### Functional relevance of NRF2 and HSF1 activation to oxidative stress-mediated apoptosis in cancer cells

Potential targets of NRF2 are mostly represented by genes encoding antioxidant proteins or enzymes (Figure 4C) to buffer the intracellular redox activities, such as *FTL*, *NQO*, *TXNRD*, *GCLM* and *GCL*. Activation of NRF2 upon the formation of oxidative signaling at the early stage enhances expression of antioxidant genes, which may consequently result in gradual ROS reduction at the intermediate stage, and moderate ROS levels at the late stage (Figure 1D). Potential targets of HSF1 are largely represented by genes encoding UPR-related chaperones (Figure 4C) such as *HSPA8*, *HSPH1*, *HSPA1A*, *HSPA9B*, *DNAJA1*, *DNAJB1*, *DNAJB6* and *SERIPINH1*. HSF1 activation and their target genes appear to be transient, providing additional evidence that UPR observed in fenretinide-treated cells occurs during the intermediate stage. Transient modulation of UPR is probably important for oxidative stress-mediated apoptosis in cancer cells, based on the fact that many of these UPR-related chaperones are functionally inhibitory to pro-apoptotic/apoptotic cascades [27]. Therefore, termination rather than preservation of UPR prior to the late stage where most apoptotic activities occur is probably essential for effective progression of apoptosis. In addition to chaperone genes, several pro-apoptotic/apoptotic genes are also regulated by HSF1, including *DEDD* [28] and *BAG3* [29]. Since the up-regulation of these genes is also terminated at the end of the intermediate stage, it is tempting to assume that they are involved in upstream activities of pro-apoptotic/apoptotic cascades.

### Substantial impact of NRF2 and HSF1 on stress-responsive transcriptome signatures relevant to oxidative stress-mediated apoptosis

**Figure 5:**
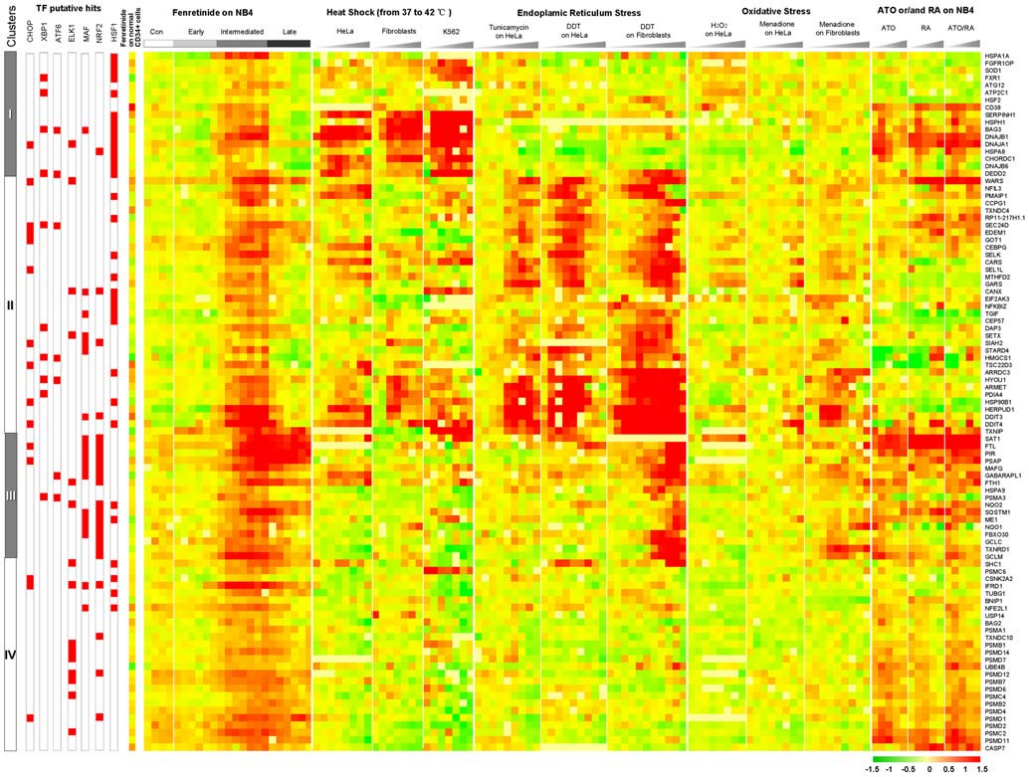
Prominent impact of NRF2 and HSF1 on transcriptome signatures underlying oxidative stress-mediated apoptosis in NB4 cells. Stress-related expression data were assembled and displayed through hierarchical clustering. TFBS information for each transcription factor is integrated on the left of the display, with putative hits marked in red. Various conditions are indicated at the top of the display. Three stress responses were all induced below the threshold where significant lethality occurred. Heat shock was induced in HeLa, fibroblast and K562 cells; Endoplasmic reticulum stress was induced in HeLa cells with the glycosylation inhibitor tunicamycin or thiol reducing agent DTT, and in fibroblasts with DTT; Oxidative stress was induced in HeLa cells with H_2_O_2_ or with menadione, and in fibroblasts with menadione. ATRA, ATO and ATRA/ATO-combined treatments induce differentiation, apoptosis, and differentiation/apoptosis in NB4 cells, respectively. Also shown is relative expression of genes in normal hematopoietic CD34^+^ cells with fenretinide treatment compared to untreated control.

Fenretinide-induced apoptosis in cancer cells occurs in response to oxidative stress, and is orchestrated by stress-responsive transcription factors, as highlighted by the modulation of a large number of stress-responsive genes. Typically, these stress-responsive genes are represented by those clustered in Group 6 (Figure 2A). Accordingly, we speculated that genes in Group 6 might represent a signature spectrum characteristic of cancer cells undergoing oxidative stress-mediated programmed cell death rather than survival upon stress stimulus. To validate this assumption, and to evaluate the potential impact of NRF2 and HSF1 on the assumed signature spectrum, we comparatively overlapped genes in Group 6 with several sets of expression data relevant to various stress responses under non-apoptotic conditions [30] and our previously published expression data relevant to ATO/RA-induced differentiation/apoptosis of NB4 cells [18]. Through hierarchical clustering followed by integration of genomic TFBS information, stress-responsive transcriptome features under apoptotic or non-apoptotic conditions were displayed (Figure 5). By comparing these features across all the conditions, the signature spectrum can be further partitioned into four categories (I–IV). Modulation of genes in category I is attributed largely to HSF1 activation, as also indicated by prominent up-regulation under heat shock. HSF1 activation under non-apoptotic heat shock conditions appears to be sustained rather than transient. Modulation of genes in category II appears to be more complex, probably because they are orchestrated by multiple stress-responsive transcription factors such as CHOP and XBP1, as implicated by the observed multifaceted TFBS composition. Our data suggest that this gene category is also involved in the ER stress/UPR occurring at the intermediate stage of oxidative stress-mediated apoptosis, based on expression patterns as well as functional annotations. Genes in category III are those directly involved in redox signaling during the intermediate and late stages, as highlighted by significant enrichment of NRF2 and its co-factor MAF. Activation of genes encoding subunits of the proteasome apparatus is one of the most prominent features in this study. These genes are exclusively clustered in category IV. TFBS analysis implicates that genes in this category are modulated by ELK1.

## Discussion

Cancer cells possess unique features not found in normal cells, which can be exploited for therapeutic design. The oxidative microenvironment of malignant cells is of particular interest. Cancer cells are often sensitive to pharmacological agents that affect the intracellular redox balance, favoring the genesis of oxidative stress and subsequent cell apoptosis. To understand how oxidative signaling is converted into programmed cell death and to develop more sophisticated protocols preferentially targeting cancer cells with minimal cytotoxicity to normal cells, we performed a comprehensive analysis of oxidative stress-mediated apoptosis in leukemia-derived NB4 cells sensitive to fenretinide apoptotic effect through a systems approach integrating methods of experimental and computational biology together with robust tools of data mining.

**Figure 6:**
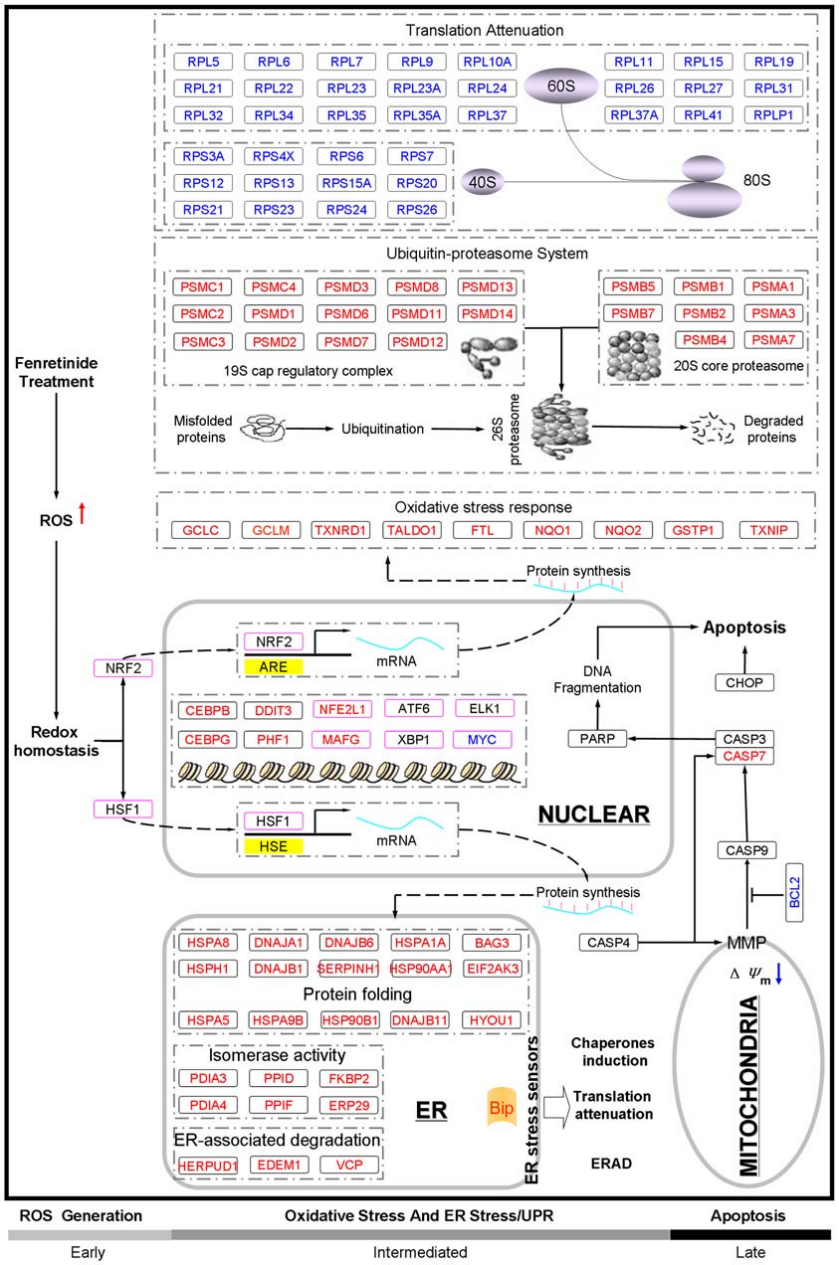
Ideogram illustrating temporal-spatial relationships among major stress-responsive events relevant to oxidative stress mediated apoptosis in NB4 cells. Early, intermediate and late stage apoptosis and their characteristic events are indicated on the bar underneath. Genes/proteins up-regulated are marked in red, and those down-regulated are marked in blue. Genes/proteins regulated at levels other than the transcriptional or translational level are marked in black. Computationally predicted transcription factors are framed in pink. Relevant cellular compartments are also indicated in the ideogram.

In this study, we have delineated a global network with the temporal-spatial relationships at both the transcriptional and functional levels using a systems approach integrating experimental and computational biology. Our results depict a typical process of oxidative stress-mediated apoptosis in cancer cells wherein stress-responsive transcription factors play prominent roles in the configuration of the underlying molecular networks (Figure 6). The rapid generation of ROS at the early stage is probably a biochemical process with minimal involvement of transcriptional regulation. When ROS reaches a certain threshold level, stress-responsive transcription factors appear to be responded. Nuclear translocation of NRF2 and subsequent induction of its target genes via antioxidant response element (ARE) may function to buffer oxidative stress response during the intermediate and late stage, whereas nuclear accumulation of HSF1 and thus activation of its target genes via heat shock element (HSE) may contribute to the occurrence of ER stress/UPR at intermediate stage, as implicated by induction of ER-localized chaperones, repression of protein translation and enhancement of ubiquitin-proteasome system. The sequential involvement of ER stress/UPR and mitochondrial associated apoptosis at the late stage is implicated separately by induction of pro-apoptotic ER stress marker CHOP and ER stress-specific caspase CASP4, and disruption of mitochondria transmembrane potential (ΔΨm) and activation of caspase cascades. Moreover, we provide solid evidence that oxidative stress is translated into downstream effects through stress-responsive transcription factors as highlighted by NRF2 and HSF1. Activation of NRF2 and thus its target genes may therefore contribute to reduction of ROS levels, as observed during the intermediate and late stage, whereas activation of HSF1 and thus its target genes may contribute to the transient occurrence of UPR. Although detailed relationships between UPR and subsequent cell apoptosis remain to be clarified, UPR termination prior to the late stage is probably essential for the effective activation of apoptosis. Notably, dynamic changes in ROS levels are of well relevance to not only the initiation of pro-apoptotic CHOP activities, but also to expression pattern of NRF2-regulated oxidative stress genes and HSF1-regulated ER stress genes during fenretinide-induced apoptosis (Supplementary Figure S2). Although these two sets of stress-responsive genes are individually considered as regulators of cellular defense mechanisms, their coordinated regulation in such manner as consistent activation of NRF2 targets and transient activation of HSF1 targets can be critical for the effective progression of apoptosis in response to fenretinide stimuli. Although exploring whether blocking specific transcription factors affects apoptosis is straightforward, our comparative transcriptome data of various stress responses clearly show that stress-relevant transcriptome features of fenretinide-treated NB4 cells can be recognized as a signature spectrum characteristic for oxidative stress-mediated apoptosis in cancer cells. Moreover, our evidence indicates that the temporal-spatial coordination of NRF2 and HSF1 in gene regulation plays an essential role in the configuration of this signature spectrum.

Choosing fenretinide as a ROS-inducing agent in cancer cells may have a number of advantages over other commonly-known oxidative stressors, such as hydrogen peroxide (H_2_O_2_) or ATO. H_2_O_2_ or ATO may cause extensive cellular damage while exerting biological functions through selective signaling molecules. However, fenretinide has shown to increase intrinsic ROS levels with minimal *in vivo* side effects, probably by interacting with specific cell membrane or cytoplasmic receptors with minimal impacts on other non-related molecules [31]. Patients undergoing long-term clinical trials with fenretinide treatment exhibit minimal side effects including impaired night vision adaptation and dry skin, which readily disappear after treatment cessation [32]. Fenretinide is more effective, and possibly more specific in inducing transcriptome changes relevant to oxidative stress-mediated apoptosis compared to oxidative stressors such as ATO (Figure 5). Our previous work has characterized the transcriptome features of ATRA-induced differentiation and ATO-induced apoptosis in NB4 cells [18], and the results of the present study have allowed us to perform a detailed comparison of the expression patterns between these three compounds. Comparing the transcriptome between ATRA-induced differentiation and fenretinide-induced apoptosis in NB4 cells, we found that fenretinide treatment series were dissimilar to ATRA treatment series, possibly reflecting the distinct biological action of these two retinoids (Supplementary Figure S3). Among our assumed signature spectrum characteristic for oxidative stress-mediated apoptosis, however, overlaps of genes between ATO-induced apoptosis or ATRA/ATO-induced differentiation/apoptosis and fenretinide-induced apoptosis in NB4 cells were not trivial (Figure 5). Based on these observations, we can speculate that fenretinide functions more in an ATO-like manner to trigger apoptosis, rather than in an ATRA-dependent manner to induce differentiation, although the possibilities that some ATRA-like indirect effects in oxidative stress-mediated apoptosis can not be excluded.

Choosing fenretinide as an anti-tumor agent may also be superior to the conventional chemotherapeutic agent, cytosine arabinoside (Ara-C), in terms of the cytotoxicity. To evaluate the potential cytotoxicity of fenretinide and Ara-C to normal cells, we isolated normal hematopoietic CD34^+^ cells from four non-leukemic donors (i.e., ND1, ND2, ND3, and ND4). As illustrated in Supplementary Figure S4, the viability (marked by both Annexin V-FITC and 7-ADD negative) was almost unchanged between 2.5 µM fenretinide-treated and untreated cells, whereas the viability after the treatment of 2.5 µM Ara-C was considerably reduced. As summarized in Table 1, normal hematopoietic CD34^+^ cells were highly resistant to fenretinide, even at the higher dose. Moreover, no significant difference was observed with the increase of fenretinide concentration, with the relative viability of 97.8±5.4% at 2.5 µM and 94.2±6.2% at 5 µM (P = 0.414). In contrast, the relative viability of CD34^+^ cells in Ara-C treated samples (43.0±10.6%) were significantly lower than that in samples treated by fenretinide of the same concentration (P = 9.04×10^−5^). These results, together with the high sensitivity of leukemia-derived cell lines NB4, U937, and HL60 to fenretinide (Supplementary Figure S1), demonstrated that fenretinide, unlike the conventional chemotherapeutic agents, could specifically target cancer cells while maintaining its minimal cytotoxicity to normal cells. Since genes in Group 6 represent a fenretinide-induced signature characteristic of cancer cells undergoing oxidative stress-mediated apoptosis (Figure 2A and Figure 5), we speculate that those genes in expression should also reflect the differences between tumor and normal cells in responses to fenretinide. To such end, we generated transcriptome profile of normal hematopoietic CD34^+^ cells with and without fenretinide treatment, and compared it with genes in Group 6 exclusively induced by fenretinide in NB4 cells. As revealed by gene set enrichment analysis (GSEA) [33], we observed significant differences in expression of these genes between fenretinide-treated NB4 cells and fenretinide-treated normal CD34^+^ cells (Supplementary Figure S5A). Rather than the coordinated induction of genes in Group 6 in fenretinide-treated NB4 cells, those genes were predominantly inactive in fenretinide-treated normal CD34^+^ cells, suggesting the absent responses of normal cells to fenretinide. As shown in Supplementary Figure S5B, similar results were also obtained by GSEA of those genes in Figure 5. Collectively, the presence of oxidative stress-responsive apoptotic signature could account for the specificity of fenretinide in targeting cancer cells while sparing normal cells.

**Table 1:** Relative viability of normal hematopoietic CD34^+^ cells isolated from 4 donors in response to fenretinide or Ara-C compared to untreated control.

Normal donors	Relative viability (%)			
	2.5 µM Ara-C	2.5 µM fenretinide		5 µM fenretinide
**ND1**	50.2	99.3		99.2
**ND2**	36.8	90.2		85.5
**ND3**	53.5	102.9		94.3
**ND4**	31.4	98.9		97.9
**Mean±SD**	43.0±10.6	97.8±5.4		94.2±6.2
**P-value (Student's test)**	9.04×10^−5^		4.14×10^−1^	

Fenretinide is known as a remarkable chemopreventive agent, and clinical data have provided evidence that it can significantly reduce the risk of second breast cancer in premenopausal women, and may be capable of eliminating cancer cells at early stages [34]. Elevated ROS generation seems to be associated with cancer cells and with early stages cancer cells. It has recently been reported that oncogenic transformation of epithelial cells causes ROS accumulation, which renders the cells sensitive to a chemopreventive natural compound that can preferentially increase ROS generation and cause apoptosis in cancer cells [35]. Furthermore, recent studies have shown that early-lineage leukemic cells rather than normal hematopoietic cells are sensitive to ROS-generating agents [36]. Accordingly, it is of significant value to further evaluate whether fenretinide exerts apoptotic effects on cancer cells at early stages. Of note, proteasome inhibitors appear to be another category of agents that can induce apoptosis in early-lineage leukemic cells [37]. In this study, we have clearly demonstrated that the proteasome inhibitor MG132 induces leukemia cell apoptosis synergistically with fenretinide. More importantly, as demonstrated in this study, deciphering the mechanistic links among different stress-responsive events may provide a broader view of oxidative stress-mediated apoptosis in cancer cells. This information may allow us to eventually develop more sophisticated protocols specifically targeting cancer cells and perhaps cancer cells at early stages. Our results identifying NRF2 and HSF1 as prominent mediators of oxidative signaling is of particular interest. Whether early stage cancer cells possess similar mechanisms under fenretinide treatment remains to be elucidated.

## Methods

### Evaluations of growth inhibition, cell apoptosis, and ROS accumulation in fenretinide treated leukemic cells

Leukemia-derived cell lines NB4 [38], U937 [39] and HL60 [40] were cultured in RPMI-1640 medium supplemented with 10% fetal bovine serum (Gibco). Fenretinide was purchased from Sigma and dissolved in ethanol at a 10 mM stock solution. Cell viability was evaluated by MTT assay, and growth inhibition was determined by the number of viable cells in treated samples compared to untreated samples. For a treatment time series, NB4 cells were treated with 1 µM fenretinide and harvested at 0, 0.5, 1, 2, 4, 6, 12, 24, 48, and 72 hours. Mitochondrial membrane potential (MMP, Δ_Ψm_) was evaluated through rhodamine 123 and propidium iodide (Sigma, St. Louis, MO) staining, followed by flow cytometry analysis. Apoptosis was determined using an ApoAlert Annexin V staining kit (Clontech) and followed by flow cytometry. To evaluate ROS changes during the time course, samples were stained with 10 µM DCFH-DA in the dark for 20 min at 37°C [31].

### Transcriptome analysis of fenretinide-induced apoptosis in cancer cells

#### Array hybridization, topology-preserving gene selection through self-organizing map (SOM) integrated singular value decomposition (SVD), and gene clustering by component plane presentation (CPP) integrated SOM

Fenretinide treated NB4 cells were harvested at 0.25, 0.5, 1, 2, 4, 6, 8, 10, 12, 15, 18, 21, 24, 30, 36, 42, 48, 54 and 60 hour, and followed by RNA preparations. Simultaneously, untreated NB4 cells were also collected after 0, 8, 12 and 15 hours in culture. Array fabrication, RNA isolation, labeling, and hybridization were performed as previously described [18], [19]. After hybridization, data acquisition was conducted using a laser scanner (Axon) and then normalized by intensity-dependent global Lowess regression. A primary expression matrix with expression values (log ratios with base 2) of 8,044 analyzable cDNA elements across 23 samples was constructed and subsequently applied to a topology-preserving gene selection procedure [41]. The selection procedure consists of three major steps: SOM transformation, SVD decomposition followed by feature recognition, and gene selection based on false discovery rate (FDR) estimation (see Methods S1). Under the FDR of 0.09, 228 neurons representing 3,345 regulated genes were selected. For gene clustering and visualization, those well-selected genes were subjected to CPP-SOM [17]–[19], [42] with 50 (10×5) neurons (see Data S1).

#### Transcription factor binding site (TFBS) enrichment and functional annotation studies

Two major steps were involved in TFBS enrichment studies: transcriptional regulatory signature database (TRSD) construction and statistical evaluation of TFBS enrichment. For the construction of TRSD, putative promoter sequences for about 25,000 RefGene reference sequences, each spanning 2-kb upstream and 200 bp downstream of putative transcription start site, were extracted from the UCSC genome browser (March 2006 build) and mapped to Entrez Gene (NCBI) for unique gene identification. These putative promoter fragments, representing 18,284 unique genes, were then scanned for putative TFBSs by a position weight matrix (PWM)-based MATCH software program [43]. Through a rank-based threshold of maximal 2,500 hits per PWM, a genome-wide TRSD was constructed, containing 493 transcriptional regulatory signatures/PWMs with the average of 1,686 potential target genes per PWM. Using this TRSD, we assessed each neuron for the significant enrichment of each transcriptional regulatory signature under the hypergeometric distribution. Under this distribution model, we calculated neuron-specific *p-values* against each regulatory signature using the entire EntrezGene as the background, with each *p-value* representing the likelihood of a specific signature enriched in the neuron. Afterward, the Benjamini-Hochberg (BH) derived step-up procedure of FDR was applied to account for multiple hypothesis testing. The calculations are summarized as follows: let the observed raw *p-values* be 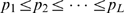 (L = 493), then the *q values* corresponding to the BH procedure is:
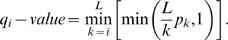



Accordingly, *q values* were utilized to determine the significance of the PWM enrichments per neuron.

For functional annotation of genes clustered in each neuron, we utilized the database of Gene Ontology (GO) (ftp://ftp.ncbi.nih.gov/gene/DATA/gene2go.gz). We carried out similar enrichment analyses through the hypergeometric distribution and followed by BH step-up procedure for *q values*, as described above.

#### Comparison of stress-related transcriptome features in apoptotic and non-apoptotic conditions

Several previously published sets of stress-related expression data under non-apoptotic conditions [30] and ATO/RA-induced differentiation/apoptosis expression data [18] were subjected to our SOM-SVD gene selection procedures. After gene selection, those genes which overlapped with Group 6 (Figure 2A) were further utilized for significant TFBS recognition in the promoter regions using our TRSD. Overlaps were organized through hierarchical clustering and followed by integration of putative hits of those significantly enriched transcription factors (i.e., HSF1, NRF2, MAF, ELK1, ATF6, XBP1 and CHOP), revealing four distinct categories (I–IV) with characteristic TFBS compositions (see Data S2).

### Cellular and molecular validations of features recognized through transcriptome analysis of oxidative stress-mediated apoptosis in cancer cells

Western blot analyses were performed using specific antibodies for GRP78 (Santa Cruz Biotechnology, Santa Cruz, CA), GADD153/CHOP/DDIT3 (Abcam, Cambridge, MA), CASP4 (BD Biosciences Pharmingen, San Diego, CA), CASP9 (Cell Signaling Technology, Beverly, MA), CASP3 (Cell Signaling Technology, Beverly, MA), PARP (Santa Cruz Biotechnology, Santa Cruz, CA), NRF2 (Santa Cruz Biotechnology, Santa Cruz, CA), HSF1 (Santa Cruz Biotechnology, Santa Cruz, CA) and ACTIN (Oncogene, Fremont, CA) on either total protein lysates or nuclear extracts. For ROS antagonist assays, NB4 cells were treated with 1 µM fenretinide and/or 100 µM of the antioxidant vitamin C (ascorbic acid sodium salt; Sigma) for 24 hours, and then subjected to apoptosis evaluation. For proteasome antagonist assays, NB4 cells were treated with 0.5 µM fenretinide and/or 0.2 µM of the proteasome inhibitor MG132 (Calbiochem) for 48 hours prior to apoptosis evaluation. For cellular localization analysis, NRF2 and HSF1 were visualized by immunofluorescence microscopy. Chromatin immunoprecipitation (ChIP) was carried out using antibodies against NRF2 and HSF1, as described previously [27], [44]. Immunoprecipitates were subjected to quantitative real-time PCR to validate potential TFBSs in gene promoters computationally which were identified by integrative analysis, as described in the previous sections.

### Evaluation of cytotoxicity in fenretinide-treated normal hematopoietic CD34^+^ cells

Fresh bone marrow cells were obtained from 4 normal donors (i.e., ND1, ND2, ND3, and ND4; see Table 1) with informed consent according to the Declaration of Helsinki and approval of Institutional Review Board at Ruijin Hospital affiliated to Shanghai Jiao Tong University School of Medicine. CD34^+^ cells were isolated using EasySep® Human CD34 Positive Selection kit (Stem Cell Technologies, Vancouver, BC, Canada) according to the manufacturer's instructions, and then were separately treated with 2.5 µM and 5 µM fenretinide, and also with chemotherapeutics as comparisons, 2.5 µM cytosine arabinoside (Ara-C; Sigma, St. Louis, MO). After 24 hours of treatment or un-treatment, the cells were then subjected to double-labeling with 7-aminoactinomycin (7-AAD; Molecular Probes, Eugene, OR) and AnnexinV-FITC (BD Pharmingen, San Diego, CA), followed by flow cytometry analysis. Viable cells were counted from both Annexin V-FITC and 7-ADD negative (i.e., no measurable apoptosis). The relative viable was calculated as viable cells with the treatment divided by those viable cells without the treatment.

### Transcriptome profiles of normal hematopoietic CD34^+^ cells with and without fenretinide treatment, and compared with stress-responsive transcriptome signature induced by fenretinide in NB4 cells

Total RNAs of CD34^+^ cells from a normal donor (i.e., ND1) treated with 1 µM fenretinide or left untreated for 12 hours were amplified and labeled with biotin according to the standard Affymetrix® protocol. The fragmented, biotinylated cDNA was then subjected to hybridization with the GeneChip® Human Genome-U133 plus 2.0 array (Affymetrix, Santa Clara, CA). Raw expression data were normalized using Affymetrix MAS 5.0 algorithm in R (Bioconductor). Detection call-based filter [45] was applied to remove all the probesets whose expression values were consistently below an empirically-determined value of minimum sensitivity, which were evaluated according to the 95^th^ percentile of all the ‘Absent’ call-flagged signals of the entire dataset. Following the normalization and filtering, gene set enrichment analysis (GSEA) [33] was utilized to determine the degree to which genes in Group 6 (Figure 2A) as well as genes in Figure 5 are overrepresented at the top or bottom of ranked gene lists from highest to lowest expression in normal hematopoietic CD34^+^ cells with fenretinide treatment compared to those without fenretinide treatment.

### Publicly deposited microarray data

The microarrays used to generate time-series transcriptome profilings of fenretinide-treated versus untreated NB4 cells are in accordance with MIAME guidelines, and are available at the Gene Expression Omnibus (GEO, http://www.ncbi.nlm.nih.gov/geo/) public database under accession number GSE16578.

## Supporting Information

Methods S1 More detailed materials related to data mining in this study
(0.07 MB DOC)

Figure S1 Cellular and molecular characterization of fenretinide-induced apoptosis in leukemia-derived NB4, HL60, and U937 cells. (A) Viable cells rate occurring upon the 48 hours treatment of a series of fenretinide concentration. (B) Loss of mitochondrial membrane potential in response to fenretinide treatment, as determined through rhodamine 123 and propidium iodide (PI) double staining, and followed by flow cytometry analysis. (C) Effects of fenretinide on ROS generation after the treatment of 12 hours, as evaluated by DCFH-DA staining prior to analysis by flow cytometry. Each point is the mean of three experiments±SD.
(0.63 MB TIF)

Figure S2 Dynamics of intracellular ROS level, pro-apoptotic CHOP mRNA, and expression pattern of NRF2-regulated-oxidative-stress genes and HSF1-regulated-ER-stress genes during fenretinide-induced apoptosis. Changes of intracellular ROS are displaying left-skewed bell-shape curve (in blue). The accumulation of ROS at the early stage (within 6 hours) not only accounts for the biological mechanism of fenretinide, but also initiates stress-inducible pro-apoptotic transcription factor CHOP activities (in light purple) and incurs the stress-responsive events, as highlighted by activation of stress-responsive transcription factors NRF2 and HSF1 and the subsequent modulation of NRF2-regulated-oxidative-stress targets (curve in cyan) and HSF1-regulated-ER-stress targets (curve in dark purple). The average expression pattern of NRF2 targets and HSF1 targets are plotted based on genes in left panel and right panel of Figure 4C, respectively.
(0.90 MB TIF)

Figure S3 Illustration of the transcriptome changes in fenretinide-treated series and ATO or/and ATRA-treated series in NB4 cells by component plane presentation integrated self-organizing map (CPP-SOM). HPR: fenretinide; ATO: arsenic trioxide; ATRA: all-trans retinoic acid.
(3.92 MB TIF)

Figure S4 Representative flow cytometry dot plots illustrating the effects of fenretinide and Ara-C on normal hematopoietic CD34+ cells. Also shown is the proportion of those viable cells marked with both Annexin V-FITC and 7-ADD negative (i.e., no measurable apoptosis).
(0.31 MB TIF)

Figure S5 GSEA analysis of (A) genes in Group 6 (Figure 2A) and (B) genes in Figure 5 regarding to transcriptome profiles of normal hematopoietic CD34+ cells with fenretinide treatment compared to those without treatment. In the contrast to coordinated induction in fenretinide-treated NB4 cells, those genes were predominantly inactive in fenretinide-treated normal CD34+ cells.
(0.52 MB TIF)

Data S1 Gene expression matrix (3,345×23) identified by SOM-SVD and analyzed by CPP-SOM in fenretinide-induced apoptosis of NB4 cells.
(2.03 MB XLS)

Data S2 Overlaps among stress-related genes between apoptotic and non-apoptotic conditions with characterersitic TFBS information.
(0.23 MB XLS)
