# Supplementary material for: Converting Redox Signaling to Apoptotic Activities by Stress-Responsive Regulators HSF1 and NRF2 in Fenretinide Treated Cancer Cells
Source: PLoS One. 2009 Oct 21;4(10):e7538. doi: 10.1371/journal.pone.0007538 (PMC2760443; doi:10.1371/journal.pone.0007538)
Supplement: Methods S1 — More detailed materials related to data mining in this study (0.07 MB DOC) [file pone.0007538.s001.doc]

**Supplementary Methods**

- **Topology-preserving gene selection through self-organizing map (SOM) integrated singular value decomposition (SVD)**

To avoid artifacts while retaining the biologically meaningful genes inherent to primary expression data, we have proposed a topology-preserving approach for gene selection, integrating SOM functionality in pre-processing[1] and SVD in pattern recognition[2]. The gene selection procedure consists of three major steps: SOM transformation, SVD decomposition followed by feature recognition, and gene selection based on false discovery rate (FDR) estimation.

1. Firstly, primary gene expression data is nonlinearly transformed into output matrix using SOM with Epanechikov (EP) kernel function, which allowed both local clustering and global preservation of topological structure.
2. Secondly, the resulting output matrix is linearly decomposed by SVD for pattern recognition. In parallel, the output matrix is randomly permuted in both row and column directions, and then decomposed by SVD to generate a set of background variables. By comparing the relative contribution of each feature variable with that of each random variable to the overall variation, the dominant feature variables are selected to form SVD subspace. Projection of the output matrix and its randomized matrix onto the same SVD subspace resulted in two different distributions of data points. The distribution resulted from the output matrix tended to be more away from the origin of the space than that resulted from the randomized matrix. Neuron-specific distance statistic is constructed by calculating the Euclidian distance of each data point to the origin of the subspace.
3. Thirdly, FDR for each neuron is assessed through multiple tests rooted in constructed distance statistic. Thus, significant neurons representing genes with characteristic patterns are selected to control the FDR at the given endurable level.

- **In-depth transcriptome analysis of fenretinide-induced apoptosis in NB4 cells using SOM-SVD and component plane presentation (CPP) integrated SOM**

1. The primary expression matrix (8,044 analyzable cDNA elements across 23 samples) was subjected to SOM-SVD for gene selection. Under FDR of less than 0.09, 228 neurons representing 3,345 regulated genes (represented by EntrezGene) were selected and subsequently used for analysis.
2. For gene clustering and visualization, the well-selected gene expression data (3,345×23) were subjected to CPP-SOM[3?]. Specifically, a software package of SOM implemented with the Matlab 6.5 environment was utilized for SOM training with 50 (10×5) neurons using EP kernel function. Illustration of the SOM outputs was visualized by CPP, depicting global transcriptional changes for each of the samples during fenretinide-untreated/treated process. By direct comparisons of transcriptome changes within/between control series and fenretinide-treated series, the process of fenretinide-induced apoptosis can be partitioned into the early (i.e., 0.25 to 6 hours), intermediate (i.e., 8 to 24 hours) and late (i.e., 30 to 60 hours) three sequential stages at the transcriptome level. Moreover, six recognizable regions can be obtained through hierarchical clustering of neurons, showing characteristic transcriptional features. For instance, 375 genes mapped to bottom-right corner/edge areas (termed Group 6) are exclusively fenretinide-induced and thus represent the signature spectrum of oxidative stress mediated apoptosis.

- **Survey of fenretinide-induced oxidative stress mediated apoptotic signature spectrum in several sets of expression data relevant to various stresses**

We compared fenretinide-induced oxidative stress mediated apoptotic signature spectrum to various typical stress responses under non-apoptotic conditions (Heat Shock, Endoplamic Reticulum Stress, and Oxidative Stress)[6] and ATO/ATRA-induced differentiation/apoptosis expression data[4].

1. We re-analyzed both previously published temporal transcriptome profiling by applying SOM-SVD for gene selection. Under FDR of less than 0.01, 491 neurons were then selected, representing 17,524 cDNA elements which contained 8,408 unique annotated genes (represented by EntrezGene) regulated during various non-apoptotic stress responses. Similarly, with the FDR of less than 0.10, 1,249 cDNA elements were selected as ATO/RA-induced differentiation/apoptosis gene set.
2. Comparatively combined with fenretinide-induced oxidative stress mediated apoptotic signature spectrum, 95 unique genes were overlapped. By screening of Transcription factor binding site (TFBS) in promoter regions of those genes using transcriptional regulatory signature database (TRSD, see Materials and Methods for its construction), several transcription factors (represented by HSF1, NRF2, MAF, ELK1, ATF6, XBP1 and CHOP) are statistically significant. Overlaps were organized through hierarchical clustering and followed by integration of putative hits of those transcription factors, displaying four distinct categories (I-IV) with the characteristic transcription factors associated.

**References**

1. Kohonen T (2001) Self-Organizing Maps. Springer, Berlin. 501 p.

2. Alter O, Brown PO, Botstein D (2000) Singular value decomposition for genome-wide expression data processing and modeling. Proc Natl Acad Sci U S A 97: 10101-10106.

3. Xiao L, Wang K, Teng Y, Zhang J (2003) Component plane presentation integrated self-organizing map for microarray data analysis. FEBS Lett 538: 117-124.

4. Zheng PZ, Wang KK, Zhang QY, Huang QH, Du YZ, Zhang QH, Xiao DK, Shen SH, Imbeaud S, Eveno E, Zhao CJ, Chen YL, Fan HY, Waxman S, Auffray C, Jin G, Chen SJ, Chen Z, Zhang J (2005) Systems analysis of transcriptome and proteome in retinoic acid/arsenic trioxide-induced cell differentiation/apoptosis of promyelocytic leukemia. Proc Natl Acad Sci U S A 102: 7653-7658.

5. Du Y, Wang K, Fang H, Li J, Xiao D, Zheng P, Chen Y, Fan H, Pan X, Zhao C, Zhang Q, Imbeaud S, Graudens E, Eveno E, Auffray C, Chen S, Chen Z, Zhang J (2006) Coordination of intrinsic, extrinsic, and endoplasmic reticulum-mediated apoptosis by imatinib mesylate combined with arsenic trioxide in chronic myeloid leukemia. Blood 107: 1582-1590.

6. Murray JI, Whitfield ML, Trinklein ND, Myers RM, Brown PO, Botstein D (2004) Diverse and specific gene expression responses to stresses in cultured human cells. Mol Biol Cell 15: 2361-2374.
