# Supplementary figures and images for: Converting Redox Signaling to Apoptotic Activities by Stress-Responsive Regulators HSF1 and NRF2 in Fenretinide Treated Cancer Cells
Source: PLoS One. 2009 Oct 21;4(10):e7538. doi: 10.1371/journal.pone.0007538 (PMC2760443; doi:10.1371/journal.pone.0007538)

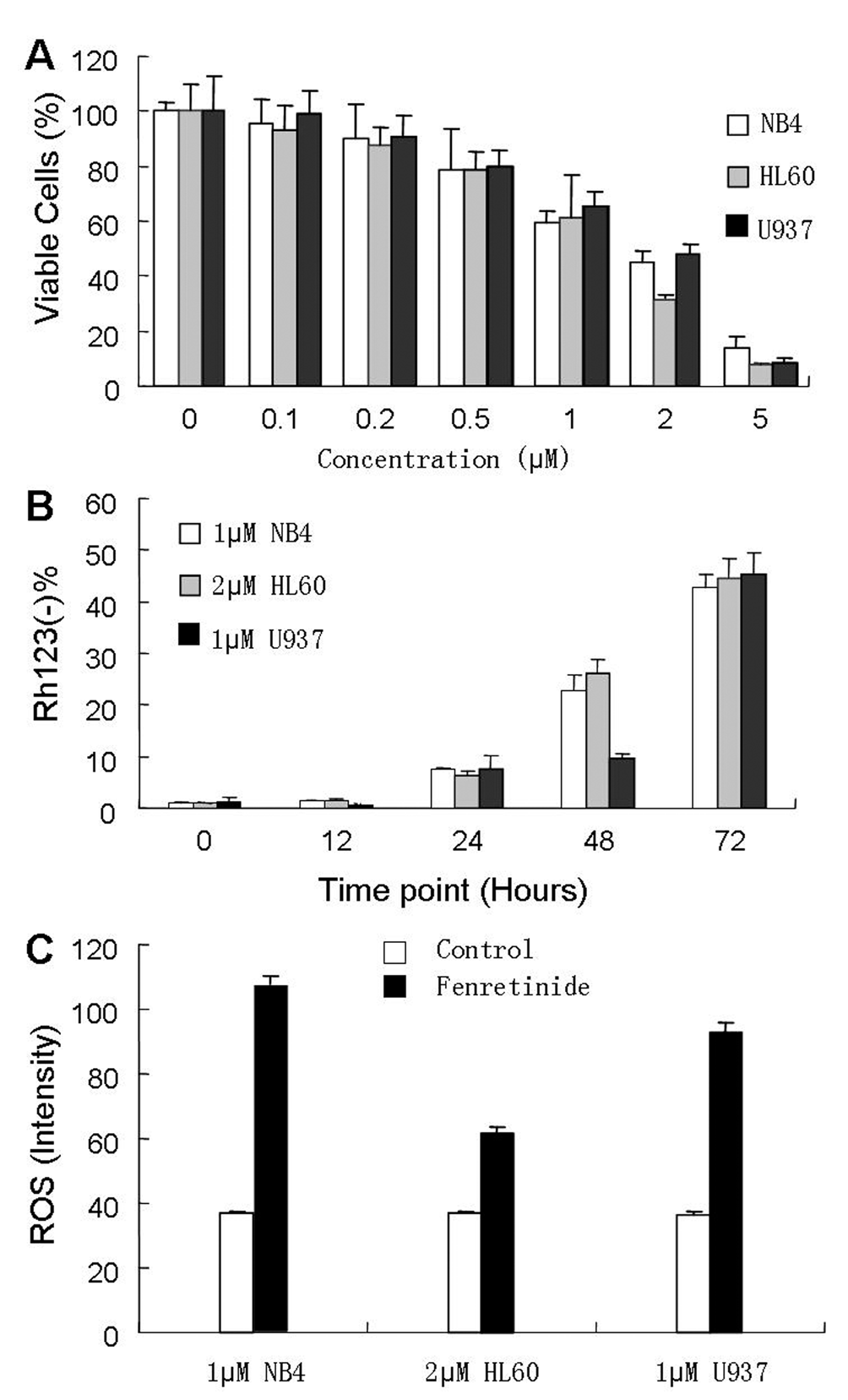

Supplement: Figure S1 — Cellular and molecular characterization of fenretinide-induced apoptosis in leukemia-derived NB4, HL60, and U937 cells. (A) Viable cells rate occurring upon the 48 hours treatment of a series of fenretinide concentration. (B) Loss of mitochondrial membrane potential in response to fenretinide treatment, as determined through rhodamine 123 and propidium iodide (PI) double staining, and followed by flow cytometry analysis. (C) Effects of fenretinide on ROS generation after the treatment of 12 hours, as evaluated by DCFH-DA staining prior to analysis by flow cytometry. Each point is the mean of three experiments±SD. (0.63 MB TIF) [file pone.0007538.s002.tif]

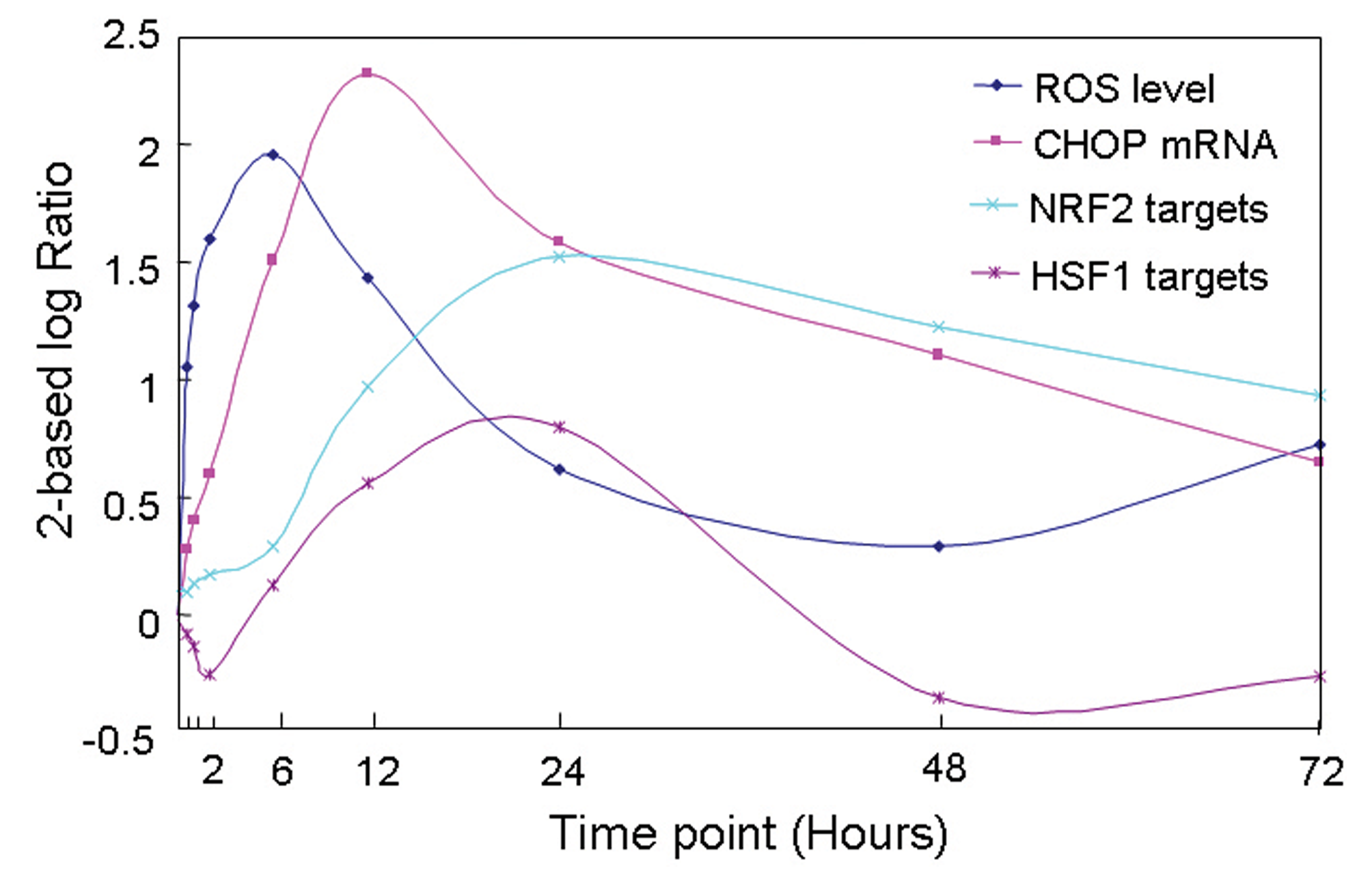

Supplement: Figure S2 — Dynamics of intracellular ROS level, pro-apoptotic CHOP mRNA, and expression pattern of NRF2-regulated-oxidative-stress genes and HSF1-regulated-ER-stress genes during fenretinide-induced apoptosis. Changes of intracellular ROS are displaying left-skewed bell-shape curve (in blue). The accumulation of ROS at the early stage (within 6 hours) not only accounts for the biological mechanism of fenretinide, but also initiates stress-inducible pro-apoptotic transcription factor CHOP activities (in light purple) and incurs the stress-responsive events, as highlighted by activation of stress-responsive transcription factors NRF2 and HSF1 and the subsequent modulation of NRF2-regulated-oxidative-stress targets (curve in cyan) and HSF1-regulated-ER-stress targets (curve in dark purple). The average expression pattern of NRF2 targets and HSF1 targets are plotted based on genes in left panel and right panel of Figure 4C, respectively. (0.90 MB TIF) [file pone.0007538.s003.tif]

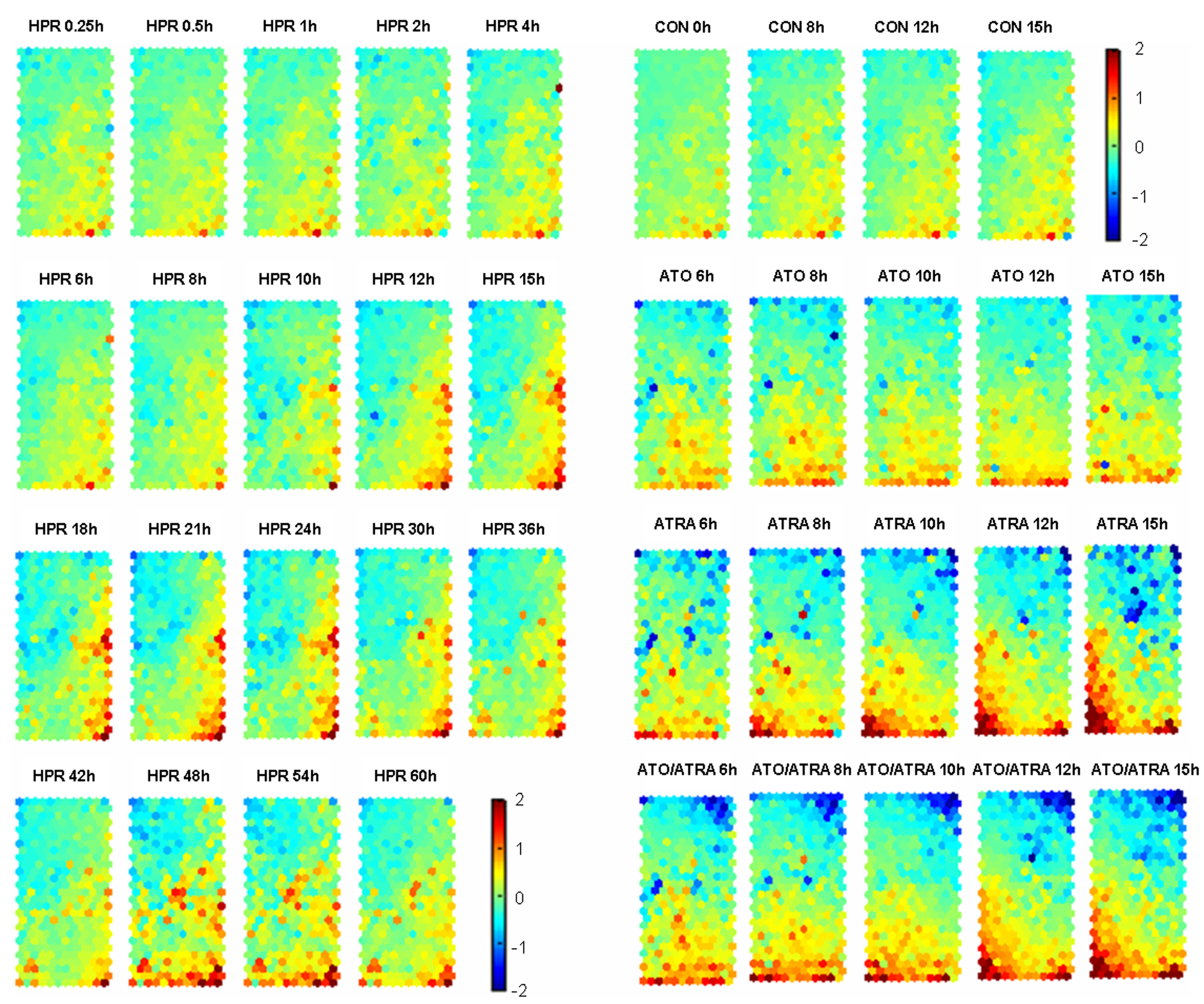

Supplement: Figure S3 — Illustration of the transcriptome changes in fenretinide-treated series and ATO or/and ATRA-treated series in NB4 cells by component plane presentation integrated self-organizing map (CPP-SOM). HPR: fenretinide; ATO: arsenic trioxide; ATRA: all-trans retinoic acid. (3.92 MB TIF) [file pone.0007538.s004.tif]

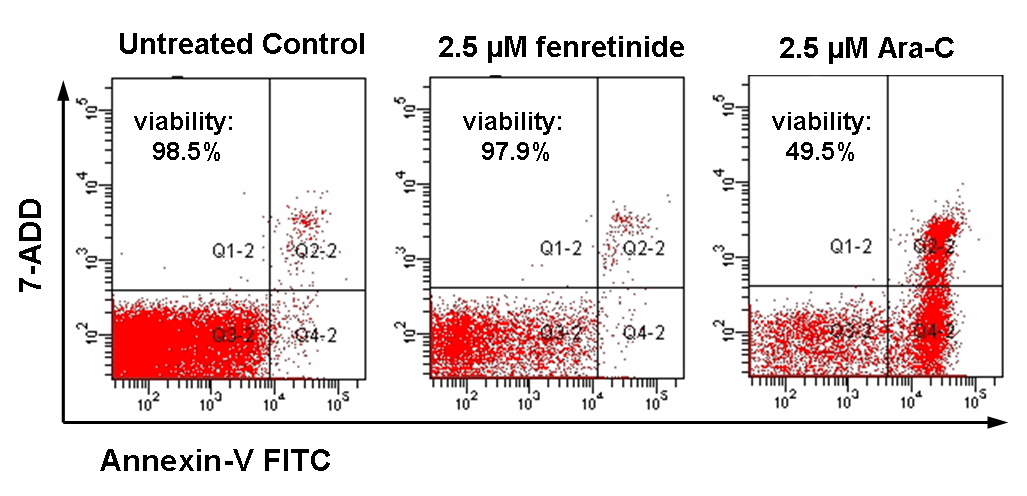

Supplement: Figure S4 — Representative flow cytometry dot plots illustrating the effects of fenretinide and Ara-C on normal hematopoietic CD34+ cells. Also shown is the proportion of those viable cells marked with both Annexin V-FITC and 7-ADD negative (i.e., no measurable apoptosis). (0.31 MB TIF) [file pone.0007538.s005.tif]

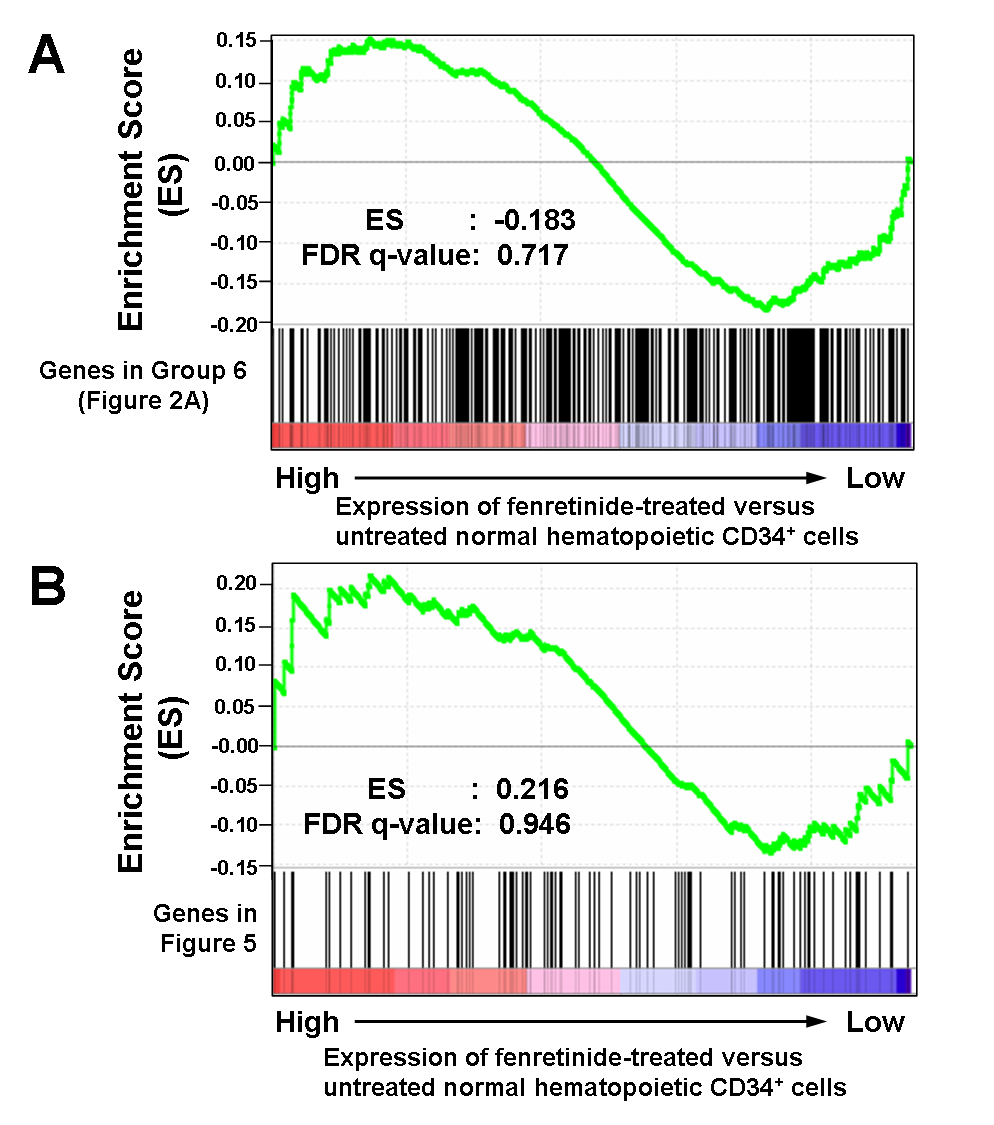

Supplement: Figure S5 — GSEA analysis of (A) genes in Group 6 (Figure 2A) and (B) genes in Figure 5 regarding to transcriptome profiles of normal hematopoietic CD34+ cells with fenretinide treatment compared to those without treatment. In the contrast to coordinated induction in fenretinide-treated NB4 cells, those genes were predominantly inactive in fenretinide-treated normal CD34+ cells. (0.52 MB TIF) [file pone.0007538.s006.tif]
